# Mortality Statistics, Chicago

**Published:** 1875-11

**Authors:** Ben. C. Miller

**Affiliations:** Sanitary Superintendent


**FOR SEPTEMBER, 1875.**

Reported by Dr. BEN. C. MILLER, Sanitary Superintendent.

CAUSES OF DEATHS.—Accident, drowning, 2; accident, by fall, 4; accident in planing mill, 2; accident, run over by wagon, 1; accident by laudanum, 1; accident by railroad, 6; accident by scalding, 1; accident by shooting, 1; angina tonsillaris, 1; aphthæ, 1; apoplexy, 4; asthma, 1; ataxia locomotor, 1; ascitis, 1; bowels, constipation of, 1; brain, congestion of, 4; brain, hæmorrhage of, 1; brain, inflammation of, 10; brain, tumor of, 1; bronchitis, 1; bronchitis, capillary, 2; cancer of breast, 2; cancer of bowels, 1; cancer of stomach, 5; cancer of œsophagus, 1; cancer of uterus, 2; cirrhosis, 1; cholera infantum, 149; cholera morbus, 4; colitis, 1; consumption, 48; convulsions, 83; croup, 8; cyanosis, 2; cystitis, 2; debility, general, 6; deficient development, 1; diarrhœa, 47; diarrhœa, chronic, 7; diphtheria, 7; dropsy, general, 2; dysentery, 14; endo-carditis, 1; enteritis,

17; entero-colitis, 7; epilepsy, 1; erysipelas, 3; exhaustion, 2; fever, congestive, 1 ; fever, intermittent, 1 ; fever, remittent, 6 ; fever, scarlet, 20 ; fever, typhoid, 46; gastritis, 3; gastro-enteritis, 5; hæmorrhagica purpura, 1; heart, disease of, 10; heart, organic disease of, 2; heart, rheumatism of, 2; heart, valvular disease of, 4 ; hepatitis, 1; hernia, incarcerated, 1; hydrocephalus, 10; inanition, 28; illeo colitis, 1; intemperance, 2; indigestion, 2 ; icterus, 3 ; jaundice, 2 ; kidneys, disease of, 1; kidneys, Bright's disease of, 5; laryngitis, 1 ; liver, atrophy of, 1; liver, congestion of, 1; liver, inflammation of, 1; lungs, congestion of, 3; lungs, scrofula of, 1; manslaughter, 1; metro-peritonitis, 1 ; meningitis, 11; meningitis, cerebro-spinal, 7; meningitis, tubercular, 4; metritis, 1; old age, 10 ; paralysis, 4 ; peritonitis, 5 ; pneumonia, 13 ; pleurisy, 1 ; phlegmonia, 1; pyæmia, 1; rheumatism, 2; scrofula, 2; septicæmia, 3; septicæmia, puerperal, 1; stomatitis, 1; suicide by drowning, 1 ; suicide by poison, 1; suicide by hanging, 1; suicide by laudanum, 1 ; syphilis congenital, 2 ; tabes mesenterica, 16 ; teething, 15 ; trismus, 2 ; ulceration of mouth and throat, 1 ; whooping cough, 19. Total, 764.

Premature births, 12 ; still births, 57. Total, 69.

Comparison.—Deaths in month of September, 1875, 764; in August, 1875, 986. Decrease, 222. Deaths in September, 1874, 809. Decrease, 45.

Ages.—Under one year, 304 ; one year to two, 148; two years to three, 30; three years to four, 8; four years to five, 8; five years to ten, 28; ten years to twenty, 35; twenty years to thirty, 56 ; thirty years to forty, 31; forty years to fifty, 30; fifty years to sixty, 29; sixty years to seventy, 23; seventy years to eighty, 18; eighty years to ninety, 6. Total, 764.

Males, 408 ; females, 356. Total, 764.

White, 756; colored, 8. Total, 764.

Married, 139; single, 625. Total, 764.

Nativities.—Atlantic Ocean, 1 ; Austria, 1 ; Bohemia, 4; Canada, 7 ; Native—Chicago, 124; Foreign—Chicago, 381; United States, other parts, 99; Denmark, 1; England, 10 ; France, 2 ; Germany, 59; Holland, 1; Ireland, 46; Norway, 3; Russia, 1; Scotland, 4 ; Sweden, 12; Switzerland, 2 ; Shetland Isles, 1 ; Wales, 1; unknown, 4. Total, 764.

Deaths daily, 25½. Mean thermometer, 61°. Rainfall, 4.39 inches.

Mortality by Wards.—Ward 1, deaths, 1 ; population in 1874, 5,725; percentage, 5,725. Ward 2, deaths, 3, population, 4,830 ; percentage, 1,610. Ward 3, deaths, 17; population, 14,861; percentage, 874. Ward 4, deaths, 14 ; population, 15,361 ; percentage, 1,097. Ward 5, deaths, 36 ; population, 20,078 ; percentage, 561. Ward 6, deaths, 75 ; population, 35,916; percentage, 479. Ward 7, deaths, 67 ; population, 31,722 ; percentage, 474. Ward 8, deaths, 46; pop. 29,143; percentage, 633. Ward 9, deaths, 43; population, 31,654 ; percentage, 720. Ward 10, deaths, 23 ; population, 17,385 ; percentage, 756. Ward 11, deaths, 23; population, 14,022 ; percentage, 609. Ward 12, deaths, 11 ; population, 16,792 ; percentage, 1,527. Ward 13, deaths, 27; population, 17,892; percentage, 663. Ward 14, deaths, 29 ; population, 16,720 ; percentage, 576. Ward 15, deaths, 132 ; population, 45,545; percentage, 345. Ward 16, deaths, 45 ; population, 21,922 ; percentage, 487. Ward 17, deaths, 42 ; population, 20,777, percentage, 495. Ward 18, deaths, 35; population, 21,392 ; percentage, 611. Ward 19, deaths, 8 ; population, 4,677 ; percentage, 585. Ward 20, deaths, 12; population in 8,995; percentage, 749.

Accidents, 18 ; County Hospital, 13 ; Foundlings' Home, 20 ; Home Good Shepherd, 2 ; Hospital Alexian Bros., 5 ; Home for Friendless, 1 ; Mercy Hospital, 4; manslaughter, 1 ; Protestant Orphan Asylum, 1 ; St. Luke's Hospital, 3 ; St. Joseph's Hospital, 3; suicide 4. Total, 764.

